# The Potential for Resident Lung Mesenchymal Stem Cells to Promote Functional Tissue Regeneration: Understanding Microenvironmental Cues

**DOI:** 10.3390/cells1040874

**Published:** 2012-10-19

**Authors:** Robert F. Foronjy, Susan M. Majka

**Affiliations:** 1 Department of Medicine, St. Luke’s Roosevelt Health Sciences Center, Antenucci Building, 432 West 58th Street, Room 311, New York, NY 10019, USA; Email: rforonjy@chpnet.org; Tel.: +1-212-523-7265; 2 Department of Medicine, Vanderbilt University, 1161 21st. Ave S, T1218 MCN, Nashville, TN 37232, USA

**Keywords:** mesenchymal stem cell, lung disease, hypertension, fibrosis, pulmonary regeneration, PDGF-BB, Wnt, sfrp

## Abstract

Tissue resident mesenchymal stem cells (MSCs) are important regulators of tissue repair or regeneration, fibrosis, inflammation, angiogenesis and tumor formation. Bone marrow derived mesenchymal stem cells (BM-MSCs) and endothelial progenitor cells (EPC) are currently being considered and tested in clinical trials as a potential therapy in patients with such inflammatory lung diseases including, but not limited to, chronic lung disease, pulmonary arterial hypertension (PAH), pulmonary fibrosis (PF), chronic obstructive pulmonary disease (COPD)/emphysema and asthma. However, our current understanding of tissue resident lung MSCs remains limited. This review addresses how environmental cues impact on the phenotype and function of this endogenous stem cell pool. In addition, it examines how these local factors influence the efficacy of cell-based treatments for lung diseases.

## 1. Introduction

**Figure 1:**
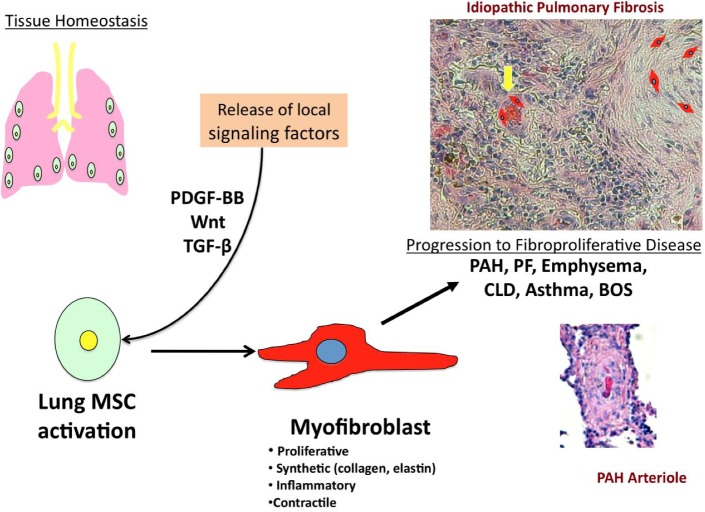
Schematic representation of the microenvironmental influences on resident lung mesenchymal stem cell function during tissue homeostasis and disease.

Many lung diseases are driven by the maladaptive proliferation of vascular and myofibroblast cells that results in dysfunctional lung remodeling. This cellular response occurs in a significantly altered pulmonary microenvironment that impedes the normal repair and regenerative capacity of tissue resident stem cells [1]. Current paradigms define the origin of these proliferative myofibroblasts as bone marrow, vascular and epithelial derived via transdifferentiation events. However, recent studies challenge this paradigm and indicate that lung MSCs are triggered by local factors to differentiate into myofibroblasts that contribute to disease progression (Figure 1). Therefore, understanding the molecular and cellular basis of endogenous lung mesenchymal stem cell (lung MSCs) participation in lung injury and repair is of critical importance. This review explores the role of the PDGF-BB and Wnt signaling pathways in the differentiation and proliferation of lung MSCSs in pulmonary diseases. Moreover, it explores the potential of manipulating these pathways to promote lung MSCs participation in functional tissue regeneration. Understanding these signaling responses may lead to new strategies that harness the reparative capacity of lung MSCs while preventing their participation in dysfunctional remodeling processes.

### 1.1. Stem Cell Therapy & Pulmonary Disease

Inflammatory lung diseases are a major cause of morbidity and mortality. There is an increasing emphasis on the development of cell-based therapies to address these conditions, but the lung is a recalcitrant candidate for these strategies because of the diverse cell types and functions. A common thread linking these diseases is the proliferation of myofibroblast cells that contribute to remodeling as opposed to repair. Because myofibroblast proliferation is a characteristic of wound healing one may suppose that proliferative lung diseases are deregulated tissue repair. Additionally, there is a pervasive lack of understanding of how chronic disease processes affect tissue resident stem cell differentiation. The functional and differentiation programs of such stem cells, is likely altered by their transformation into myofibroblasts that participate in remodeling, rather than repair. Therefore, prior to testing cell‑based therapy it is desirable to use pre-clinical animal models of lung disease to determine how changes in the lung tissue during the development of disease affect resident stem cell differentiation and function, including lung MSCs. 

### 1.2. Multipotent Resident Lung MSCs

The functionality or ‘stemness’ of stem cells is highly influenced by the local microenvironment or niche. Thus, disease processes that alter this niche impair the capacity of its resident stem cells to function, undergo self-renewal, proliferate and properly differentiate [2,3]. Adult pulmonary tissue resident MSCs demonstrate a phenotype and function similar to BM-MSCs and have been identified in the side population (SP) of cells from both murine and human lung tissue [4,5] as well as bronchoalveolar lavage fluid from human lung allografts [6]. Depending on their microenvironment, the lung MSCs demonstrate properties similar to other tissue MSCs including multilineage differentiation, paracrine anti-inflammatory properties, suppression of T cell proliferation as well as the ability to differentiate to myofibroblasts [4,5,6,7]. 

Tissue resident mesenchymal stem cells (MSCs) are important regulators of tissue repair or regeneration, fibrosis, inflammation, angiogenesis and tumor formation. Lung MSCs have been identified and characterized by using flow cytometry to detect Hoechst 33342 vital dye efflux by lung cells in combination with absence of the hematopoietic marker, CD45. *In vitro*, these Hoechst33342^dim^CD45^neg^ cells demonstrate multilineage mesenchymal differentiation potential to osteocyte, adipocyte and chondrocytes lineages and express the characteristic mesenchymal cell surface determinants ABCG2, CD90, CD105, CD106, CD73, CD44 and ScaI. In addition, they lack the hematopoietic markers c-kit and CD34 [4,5,7,8,9]. Lung MSCs also exhibit high telomerase activity which indicates the capacity for self-renewal [4,5,7]. The expression of high levels of telomerase, gives MSCs the ability to survive and replicate to generate many more offspring than typical somatic cells, similar to cancer cells [4,5,7,8,9]. These properties allow a small number of cells to contribute substantially to both tissue regeneration and to proliferative diseases [3,7,8,9,10,11,12,13,14]. 

Lama *et al.* and Hennrick *et al.* identified an additional population of MSCs derived from bronchoalveolar lavage fluid in patient allograft tissue or tracheal aspirates from ventilated neonates, respectively [6,15]. The human lung MSCs demonstrated a mesenchymal signature, multilineage differentiation to bone, fat and cartilage, myofibroblasts as well as combined expression of STRO-1, CD73, CD90, CD105, CD166, CCR2b, CD13, prolyl 4-hydroxylase and lack of CD11b, CD31, CD14, CD34 and CD45. Furthermore, MSCs isolated from tissue allograft bronchoalveolar lavage fluid also suppressed T cell proliferation [7,16,17]. 

Pulmonary arterial hypertension (PAH) results from vasoconstriction, remodeling of the large and small pulmonary arteries and occlusion of microvessels in the lung (Figure 1). The PAH mortality rate in adult is 50 percent within five years after diagnosis. PAH may be heritable and termed familial or HPAH, idiopathic and a secondary complication of many adult lung diseases such as chronic obstructive pulmonary disease (COPD/emphysema) and pulmonary fibrosis (PF). Functional studies of the Hoechst33342^dim^CD45^neg^ resident lung MSCs demonstrate that they regulate the severity of bleomycin injury via modulation of the T-cell response [7]. Jun *et al.* elegantly documented that bleomycin treatment of mice induced the loss of these endogenous lung MSCs and elicited fibrosis (PF), inflammation and pulmonary arterial hypertension. Replacement of resident stem cells by administration of isolated lung MSCs attenuated the bleomycin-associated pathology and mitigated the development of pulmonary arterial hypertension. In addition, lung MSCs modulated a decrease in numbers of inflammatory cells in bronchoalveolar lavage fluid and inhibition T cell proliferation. Gene expression analysis indicated that lung MSCs are a bona fide subpopulation of pulmonary mesenchyme, differing from lung fibroblasts in terms of pro-inflammatory mediators and pro-fibrotic pathways. These data suggest that lung MSCs function to protect lung integrity following injury however when endogenous MSCs are lost this function is compromised. Therefore, similar to other tissue resident stem cells, lung MSCs may regulate their native tissue repair. The rigorous characterization of cell surface markers facilitated the detection as well as co-localization of both murine and human lung tissue MSCs with the alveolar capillary network suggesting that these cells play a role in maintenance of the distal lung [7].

In addition to their reparative properties, several studies indicate that lung MSCs, under certain conditions; mediate pathogenic changes within the lung [18,19]. Indeed, the behavior of MSCs is highly sensitive to the microenvironment to which these cells are exposed [20]. As an example, it was recently shown that TGF-β expression within the lungs of premature infants stimulates MSCs to differentiate into myofibroblasts [21] (Figure 1). This is significant as myofibroblasts induce dysfunctional matrix remodeling that results in the development of the chronic lung disease, bronchopulmonary dysplasia which is characterized by thickened septae and decreased functionality of the alveolar-capillary surfaced for gas exchange [22]. Similar findings were observed in lung allografts from transplanted patients [23]. In this study, lung derived MSCs isolated from the airways had increased expression of type I collagen and α-smooth muscle actin and readily differentiated into myofibroblasts upon treatment with IL-13 or TGF-β [23]. Allograft survival is limited by bronchiolitis obliterans, a fibrotic constriction of the airways [24]. Those MSCs from BOS subjects had a profibrotic phenotype indicating that this cell type was an important mediator of the fibrotic changes. Thus, these findings indicate that MSCs are a critical factor in the development of dysfunctional lung remodeling in these diseases.

Aside from affecting fibrotic disorders, MSCs can affect diseases like asthma that are characterized by lung inflammation and remodeling. Asthma is an inflammatory disorder that is typified by a proliferation of airway smooth muscle cells and the accumulation of myofibroblasts in airway subepithelium [25]. These changes promote airway obstruction and render the patient less responsive to bronchodilators [26]. Lung MSCs differentiate into these cell types and the numbers of myofibroblasts are significantly increased within the lungs of asthmatic patients [27]. Moreover, lung MSCs are increased in the ova sensitization asthma model suggesting that an aerosol challenge may act through lung MSCs to promote processes that lead to the development of asthma [28]. Together, these studies demonstrate that the biological effects of MSCs are highly influenced by the microenvironment in which they reside. Understanding these effects will better enable researchers to harness the reparative and immunomodulatory properties of MSCs while preventing the pathological remodeling changes that they induce in the lung.

## 2. PDGF-BB ad Wnt: Two Key Signaling Targets in Inflammatory Lung Disease and Vascular Remodeling

Maladaptive proliferation of vascular and myofibroblast cells is a hallmark of many lung diseases. These cells, as presumably lung MSCs, respond to the pulmonary microenvironment, which is likely impairing repair and regeneration. PDGF and Wnt signaling are two key pathways involved in the “programming” of MSC.

### 2.1. PDGF-BB Signaling & Fibro-Proliferative Lung Disorders

PDGF-BB is expressed at low levels in adult lung parenchyma, endothelium, platelets and macrophages. Lung mesenchymal cells express high levels of PDGFRβ and respond to increases in PDGF-BB with proliferation and collagen production [29,30]. Its expression is up-regulated in lung parenchyma following animal exposure to low oxygen tension, hypobaric hypoxia and mechanical stress [30,31,32,33,34,35]. Similarly, PDGF-BB promotes loss of quiescence and differentiation to a proliferative myofibroblast phenotype of hepatic stellate cells [36]. Elevated levels of PDGF are evident in lung fibrosis (Figure 1), bronchiolotis obliterans pneumonia, post-transplant obliterative bronchiolitis, histiocytosis X, pneumoconiosis and fibrosis associated with PAH [29]. In PAH a layer of myofibroblasts and matrix accumulates between the EC and internal elastic lamina. The mechanisms involved in this process remain largely unknown [30]. PDGF levels also increase in lavage fluid following acute lung injury and prior to onset of symptoms in Hermansky-Pudlak syndrome [29]. Studies using *in vivo* and *in vitro* models of vascular neointimal thickening following various injuries have substantiated a role for PDGF-BB during this pathological process [37,38,39,40,41]. Inhibition of PDGFB signaling with multiple inhibitors attenuates pulmonary fibrosis and PAH by blocking fibroblast proliferation, muscularization of the vasculature, intimal thickening and matrix deposition [33,34,42,43]. However, the interpretation of these studies is limited by the varying specificity of the inhibitors. These studies elegantly link PDGF-BB signaling to the transition of the mesenchyme to a profibrotic phenotype that disrupts the normal pulmonary architecture. 

MSC derived from bone marrow constitutively express PDGF-B and the addition of exogenous protein promotes their growth and differentiation [44]. PDGF-BB receptor, PDGFRβ, is pivotal in the adhesion and migration of MSC through regulation of integrin binding to fibronectin suggesting that fibronectin rich matrix may recruit these cells to sites of remodeling or wound healing [45]. PDGF-B exposure also stimulates MSC differentiation to pericytes which then migrate to stabilize newly formed vasculature in tumors [46]. Taken together these studies provide a basis to investigate PDGF dependent mechanisms that define endogenous lung MSCs and the differentiation of other multipotent stem cells during disease in order to identify interventions and facilitate repair. 

### 2.2. Wnt Signaling in Fibro-Proliferative Lung Disorders

The Wnt family of proteins is a highly conserved group of signaling molecules important in fundamental biological process including development and repair. β-catenin protein is a central mediator of canonical Wnt signaling via coactivation of TCF/LEF transcription factors [47,48,49,50,51,52,53]. β‑catenin is regulated at the level of synthesis, activation/translocation to the nucleus and proteasomal degradation through a destruction complex (CKI and GSK kinases) [47,48,49,50,51,52,53]. Constitutive activation of the pathway due to mutations in β-catenin result in changes in cell fate specification during development and in adulthood as well as tumor formation [52]. Sustained activation of β-catenin can result in hypercellularity of tissue and deregulated self-renewal [49,53]. In BM-derived MSCs autocrine Wnt signaling regulates self-renewal and lineage specific differentiation, including osteocyte, chondrocytes and adipocyte [47,48,50,51,54]. 

Wnt signals through the canonical pathway involving β-catenin and through non-canonical pathways involving protein kinase C (PKC) [55] and MAPK8 (also known as JNK1) [56]. In the canonical pathway, binding of Wnt to its receptor triggers the translocation of β-catenin to the nucleus where it turns on gene expression by displacing the transcription inhibitor Groucho/HDAC from the T‑cell specific factor (TCF)/lymphoid enhancer binding factor-1 (LEF1). The genes induced by β‑catenin regulate MSC proliferation, differentiation [57], migration [58] and survival [59]. The non‑canonical pathways do not act through β-catenin and in some cases they actually antagonize β‑catenin signaling. Non-canonical Wnt pathways regulate the proliferation and differentiation of MSCs via Dvl or Ca^++^-dependent processes [51]. However, the effects of Wnt signaing on MSCs are quite complex. Not only can the canonical and non-canonical pathways antagonize each other but also Wnt concentration can affect signaling responses. Indeed, one study showed that low dose Wnt treatment stimulated MSC proliferation while high dose treatment inhibited it [60]. Adding further complexity to the effects of Wnts of MSCs is the presence of an elaborate network of negative regulators that block receptor binding or intracellular signaling to tightly control Wnt signaling. Wnt inhibitory factor (WIF-1) and secreted frizzled related proteins (SFRPs) bind soluble Wnts and inhibit interaction with the frizzled receptor [61] thereby antagonizing their action. In contrast, Dickkopfs (Dkks) and Sclerostins target the LRP receptors and prevent the propagation of intracellular signals through these receptors [62,63]. During osteocyte development, SFRPs regulate MSC differentiation [64] and apoptosis [65] and WIF-1 completely counteracted the Wnt3a mediated inhibition of MSC differentiation into chondrocytes [66]. Studies indicate that Dkks promote MSC proliferation while at the same time maintaining those cells in an undifferentiated state [67]. Though study in this field is just emerging, evidence indicates that the Wnt signaling pathway and its inhibitors will be key determinants of the effects of MSCs within the lung. Already studies have shown that the Wnt/ β-catenin pathway attenuates experimental emphysema [68] and the Wnt inhibitor SFRP-1 up regulates the expression of proteases that are important in the development of human emphysema [69]. Conversely, during lung fibrosis, activation of the Wnt pathway is present in proliferative myofibroblast lesions and plays an important role in the fibrotic changes that occur [70]. Further studies are needed to understand how Wnt signaling and MSCs interact to modulate the development of pathogenic changes in the lung. This promises to be a complex process to decipher as the effects of Wnts depend on the specific cell type, disease process and expression of counter regulatory inhibitory molecules. 

## 3. Conclusions

In conclusion, studies are needed to fill gaps in our understanding of how resident lung MSCs are impaired during disease and their role during the development of disease which will be useful for designing further intervention.
